# Impact of the COVID-19 pandemic on eye removal procedures at an urban level 1 trauma center

**DOI:** 10.5935/0004-2749.2024-0312

**Published:** 2025-06-24

**Authors:** Sruthi Kodali, Yael Steinberg, Afshin Parsikia, Joyce N. Mbekeani

**Affiliations:** 1 Department of Ophthalmology, Stony Brook University, Stony Brook, New York, USA; 2 Department of Ophthalmology, Boston University Medical Center, Boston, MA, USA; 3 Department of Research Services, University of Pennsylvania, Pittsburgh, PA, USA; 4 Department of Ophthalmology & Visual Sciences, Albert Einstein College of Medicine, Bronx, NY, USA; 5 Department of Surgery (Ophthalmology), Jacobi Medical Center, Bronx, NY, USA

**Keywords:** Eye injuries, Eye enucleation, COVID-19, Pandemics, Ethinicity, Inflammation, Trauma centers

## Abstract

**Purpose:**

To evaluate the changes in the rates and indications of eye removal procedures during the recent COVID-19 pandemic.

**Methods:**

The medical records of all patients who underwent eye removal from 2007 to 2022 were retrospectively reviewed. The patient demographic data and indications for surgery were collected. Data from two groups of patients (prepandemic surgery and postpandemic surgery) were compared. Statistical significance was set at p<0.05.

**Results:**

Fifty-nine patients underwent enucleation (69%), evisceration (27%), or exenteration (3%). The mean (SD) age of the patients was 55.9 (19.4) years, and most (69%) of the patients were males. Most (47%) of the study population were Black. The common indications for eye removal were trauma (41%), painful blind eye (34%), and infection/inflammation (24%). The types of trauma were assault (55%), accidental (39%), and self-inflicted (6%). The mean (SD) monthly rates of eye removal increased from 0.25 (0.50) in the prepandemic period to 0.77 (0.91) during the pandemic (p<0.001). These increases were noted in both males (p=0.003) and females (p=0.001) and were the highest among Black patients [0.42 (0.76); p<0.001]. Among the indications of eye removal, painful blind eyes [0.35 (0.75); p<0.001] and ocular trauma [0.31 (0.47); p=0.051] exhibited the greatest increases following the pandemic.

**Conclusion:**

The rate of eye removal procedures increased during the recent pandemic. Although delayed care of chronic eye conditions may have contributed to the increased rates of painful blind eyes, the increased trauma-related eye removals may be attributed to the simultaneous spike in violent assaults in New York City.

## INTRODUCTION

Several studies have demonstrated the negative impact of the COVID-19 pandemic on visual outcomes, primarily due to the delay in administering ophthalmologic care^(1^,^2)^. For example, a delay in the detection of ocular malignancies^(3^,^4)^ or treatment of chronic conditions such as macular degeneration^(5^-^7)^ during the pandemic was associated with vision-threatening consequences.

One extreme consequence of delayed care is eye removal, which is performed as a last resort for uncontrollable infections, malignancies, trauma, painful blind eyes, absolute glaucoma, certain cosmetic defects, and for preventing sympathetic ophthalmia^(8^-^10)^. The three procedures commonly used for permanent eye removal are enucleation, evisceration, and exenteration, and the choice of procedure usually depends on the ophthalmic condition and its extent. Studies conducted during the COVID-19 pandemic revealed an increase in the rate of eye removal procedures. The authors attributed these trends to late presentation for necessary eye care, resulting in the need for terminal care. These trends were mainly reported for ocular tumors^(11^,^12)^. At Wills Eye Hospital (Pennsylvania, USA), the rates of enucleation for ocular melanoma increased from 6% prepandemic to 12% postpandemic. This increase was attributed to the more advanced presentation, which was a result of delayed cancer detection^(11)^.

In our institution, we observed a similar increase in the rates of eye removal procedures, which were mainly attributed to painful blind eyes and trauma. Our institution is a Level 1 trauma center that serves approximately 8.3% of the Bronx’s population and caters to a racially diverse community^(13)^. Its patient population includes the following: Hispanic or Latino, 41%; Black non-Hispanic, 33%; White non-Hispanic, 9%; and other racial/ethnic groups, smaller percentages. These data reflect the broader diversity of the Bronx, where >57% of the residents are Hispanic or Latino and 28% are Black or African American^(14)^. During the pandemic, our institution remained open to all types of cases, including trauma and COVID-19 infections. In this study, we aimed to determine the trends in the rates and indications for eye removal surgery, before and after the COVID-19 pandemic at an urban Level 1 trauma center. To the best of our knowledge, this is the first study to report on the changes in the rates of eye removal procedures in the United States following the pandemic.

## METHODS

### Patient identification

The medical records of all patients who had undergone evisceration, enucleation, or exenteration at the Jacobi Medical Center between January 2007 and April 2022 were retrospectively reviewed. This study was approved by the institutional review board (IRB) of the Albert Einstein College of Medicine, Bronx, New York (No: 2022-13870). This study was conducted in accordance with the relevant guidelines and regulations of the Declaration of Helsinki. Furthermore, the study was compliant with the Health Insurance Portability and Accountability Act. The need for informed consent was waived due to the retrospective nature of the study and the deidentification of data for analysis). The year 2007 was chosen for expedience because electronic medical records using similar coding documentation were available from this time. Patients were identified using the following current procedure terminology codes for removal of eye contents: 65091 and 65093 for eviscerations; 65101, 65103, and 65105 for enucleations; and 65110, 65112, and 65114 for exenterations. The following patient data were collected: patient demographics (age, sex, and race/ethnicity), eye laterality, date of the procedure, indication for the procedure, and procedure type. The indications were broadly classified as trauma, infections, painful blind eyes, inflammation, and neoplasia. Patients who presented with painful eyes secondary to trauma were categorized under “trauma”. Patients who had not undergone any eye removal procedure or those with incomplete files were excluded from the analysis.

### Statistical analysis

Data was statistically analyzed and presented according to the Strengthening the Reporting of Observational Studies in Epidemiology (STROBE) guidelines for reporting epidemiologic studies. The continuous variables are presented as mean and standard deviation (SD) or median and interquartile range (IQR). The patients were divided into the following two groups: prepandemic (January 2007 to February 2020) and postpandemic (March 2020 to April 2022). The categorized variables, patient demographics and indications, number and types of eye removals, were compared using paired tests, two-tailed *t-*test, and χ^^2^^ tests. Statistical significance was set at p<0.05. Descriptive and analytical calculations were performed using STATA (version 17; StataCorp, College Station, TX, USA). Graphs and tables were constructed using Microsoft Excel and Word (Microsoft Corp., Redmond, WA, USA).

## RESULTS

Of the 356 extracted medical records, 59 were complete files of patients who had undergone an eye removal procedure between January 2007 and April 2022. Over this 15-year period, enucleation was the most common procedure (69.5%), trauma was the leading indication for eye removal (41%), and most patients (69.5%) were male with a diverse racial and ethnic representation (Table 1).

**Table 1:** Descriptive characteristics of the patients who underwent eye removal at an urban, Level 1, trauma center before and after the COVID-19 pandemic (2007-2022)

Variable	Total n=59	Prepandemic n=39	Postpandemic n=20
Mean number of cases per year	3.9	3	10
Age, years			
Mean (SD)	55.9 (19.4)	53.9 (19.4)	60.7 (21.3)
Median	56	53.5	58
Range	13-101	13-90	19-101
Sex, n (%)			
Male	41 (69.5)	28 (71.8)	13 (65)
Female	18 (30.5)	11 (28.2)	7 (35)
Race/ethnicity, n (%)			
Black	28 (47.5)	16 (41)	12 (60)
Hispanic	16 (27)	11 (28.2)	5 (25)
White	3 (5)	1 (2.6)	2 (10)
Asian	2 (3)	2 (5)	0
Other/unknown	10 (17)	9 (23)	1 (5)
Procedures, n (%)			
Evisceration	16 (27)	13 (33.3)	3 (15)
Enucleation	41 (69.5)	25 (64)	16 (80)
Exenteration	2 (3.4)	1(2.6)	1 (5)
Indications, n (%)			
Acute trauma	24 (40.7)	16 (41)	8 (40)
Painful blind eye	20 (33.9)	12 (30.8)	8 (40)
Infection	13 (22)	9 (23.1)	4 (20)
Oncological Malignancy	1 (1.7)	1 (2.6)	0
Inflammatory	1 (1.7)	1 (2.6)	0

SD= standard deviation.

The annual incidence of eye removal procedures was 2.9 during the pre-COVID period (January 2007-March 2020) and 10 during the post-COVID period (March 2020-April 2022).

**Figure 1:**
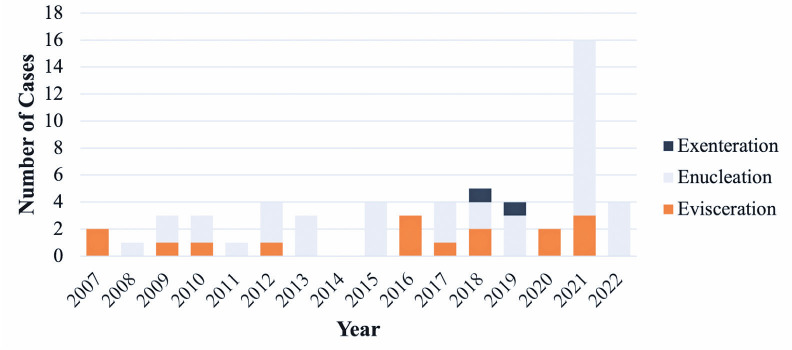
Number of eye removal procedures per year from 2007 to 2022.

The annual frequency of eye removal procedures increased from 2.9 cases during the prepandemic period to 10.5 cases in the postpandemic. Two patients underwent surgery just prior to the declaration of the USA Public Health Emergency due to COVID-19 (March 2020). The annual frequency spiked to 16 cases in 2021, which is an increase of 452% from the prepandemic average. Similarly, the four cases in 2022 represented an increase of 38% from the prepandemic average (Table 2, Figure 1).

**Table 2:** Differences in the monthly rates of eye removal before and after the COVID-19 pandemic in an urban, Level 1, trauma center (2007-2022)

Variable	Total	Prepandemic	Postpandemic	p-value
Eye removal procedures, n	59	39	20	-
Months, n	183	157	26	-
Monthly rate of eye removal procedures, mean (SD)	0.32 (0.60)	0.25 (0.50)	0.77 (0.91)	**<0.001**
Demographic data				
Age, mean (SD) Gender, mean (SD)	55.93 (19.37)	53.74 (19.34)	60.69 (21.25)	0.29
Males	0.22 (0.52)	0.18 (0.43)	0.5 (0.86)	**0.003**
Females	0.098 (0.29)	0.07 (0.26)	0.27 (0.45)	**0.001**
Race/ethnicity, mean (SD)				
Black	0.15 (0.43)	0.11 (0.33)	0.42 (0.76)	**<0.001**
White	0.04 (0.19)	0.01 (0.11)	0.19 (0.40)	**<0.001**
Hispanic	0.021 (0.146)	0.019 (0.137)	0.038 (0.196)	0.53
Common indications, mean (SD)				
Trauma (All)	0.17 (0.39)	0.15 (0.37)	0.31 (0.47)	**0.051**
Assault	0.098 (0.298)	0.08 (0.27)	0.19 (0.40)	0.083
Accidental	0.07 (0.27)	0.06 (0.26)	0.12 (0.33)	0.31
Painful blind eye	0.11 (0.39)	0.07 (0.28)	0.345 (0.75)	**<0.001**

Rates of eye removal procedures increased from 0.25 (0.50) removals per month pre-COVID 19 to 0.77 (0.91) removals per month post-COVID 19 (p<0.001). Rates of eye removal procedures due to a painful blind eye increased significantly following the start of the pandemic [0.35 (0.75); p<0.001]. Furthermore, there was also a significant increase in the rates of eye removal procedures due to trauma [0.31 (0.470); p=0.051]. P-values were calculated using two-tailed *t-*test, and χ^^2^^. P<0.05 was considered significant. Bold p-values indicate significance.

A greater percentage of eye removal procedures were performed due to a painful blind eye during the postpandemic period.

**Figure 2:**
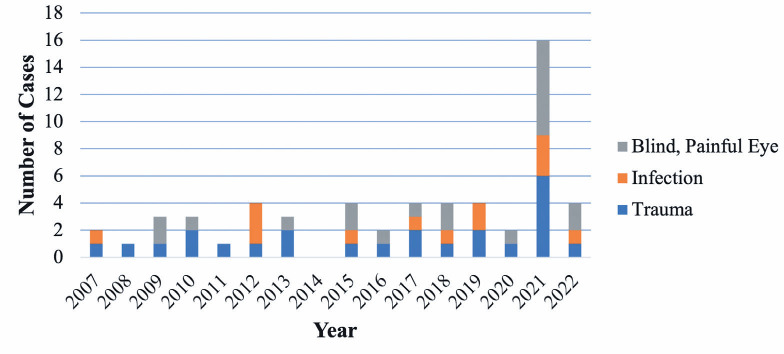
Number of eye removal procedures per year according to the indication (2007-2022).

There were no significant demographic differences between patients who underwent surgery before the pandemic and those who underwent surgery after the pandemic. Trauma and painful blind eyes were the most common indications for surgery in the prepandemic and postpandemic periods, respectively. The rate of eye removal procedures for painful blind eye increased from 30.8% in the prepandemic period to 40% in the postpandemic period. However, the rate of procedures for acute trauma remained almost the same (41% vs 40%) (Figure 2).

The annual rate of trauma-related eye removal increased from 1.4 before the pandemic to 3.0 after the pandemic, which is a 214% rise. In NYC, the annual number of violent assaults rose from 20,475 before the pandemic to 22,835 in 2021, which is a rise of 12%. In the Bronx, the rate of felony assaults surged by 23.3% (2021-April 2022), which is consistent with the 428% rise in trauma-related eye removals.

The mean (SD) number of eye removal procedures increased from 0.25 (0.50) per month in prepandemic period to 0.77 (0.91) per month in the postpandemic period (p<0.001. Both males (p=0.003) and females (p=0.001) experienced higher removal rates in the postpandemic period. Black patients exhibited the greatest increase in eye removal rates, increasing from 0.11 (0.03) in the prepandemic period to 0.42 (0.76) in the postpandemic period (p<0.001). The rates of eye removal procedures for painful blind eyes [0.35 (0.75); p<0.001] and trauma [0.31 (0.470); p=0.051] increased significantly during the postpandemic period. The rates of eye removal procedures for accidental trauma did not increase significantly (p=0.31), while those for assault trended toward significance (p=0.083) (Table 2).

## DISCUSSION

We found a significant increase in the number of eye removal procedures performed following the recent COVID-19 pandemic. This increase was primarily driven by the significant rise in prevalence of painful blind eyes and trauma during the postpandemic period. These findings highlight the impact of delayed ophthalmic care during the pandemic, which led to the progression of chronic conditions and more emergent presentations requiring eye removal.

Painful blind eyes, the most common indication for eye removal in our study, can arise due to various acute and chronic causes, including severe glaucoma, chronic retinal detachment, bullous keratopathy, uveitis, endophthalmitis, chronic hypotony, malignancy, and ocular trauma^(15^,^16)^. Trauma, the second most frequent indication for eye removal in our study, also exhibited a marked increase during the postpandemic period. Our data demonstrated a lag between the onset of the pandemic and the surge in eye removal procedures, which suggests that the delayed access to routine ophthalmic care may have exacerbated the chronic conditions, culminating in late-stage emergent cases. These results highlight the need for strategies to maintain access to ophthalmic care during times of societal stressors to prevent such outcomes.

Eye removal procedures are significantly associated with morbidity. They can cause phantom pain, worsen quality of life due to increased stress, and negatively impact self-perception and mental health^(17^,^18)^. Thus, identification of factors that can precipitate eye removal is crucial, particularly in times of stressors such as the pandemic. By identifying these factors, preventative measures can be implemented and adverse outcomes can be mitigated.

Our data contributes to the currently available literature regarding the effects of the COVID-19 pandemic on delayed medical care and suboptimal health outcomes. The effect of the pandemic on decreased number of ophthalmologic visits is well-documented^(19^,^20)^. This decrease in numbers has been attributed to government lockdowns, increased social distancing measures, and patient’s reluctance to seek medical attention^(21)^. One study by the UK Ocular Oncology Services demonstrated that the number of uveal melanoma cases diagnosed during the national lockdown had decreased by 43%, resulting in an increased number of patients presenting with more advanced ocular diseases after the pandemic. Furthermore, they attributed the increase in advanced ocular diseases after the pandemic to the fact that uveal melanoma is often incidentally detected during routine ophthalmic care for other ocular comorbidities^(12)^. Similarly, in the study conducted at Wills Eye Institute, patients presented with larger and more advanced melanomas after the pandemic, with the rates of enucleation nearly doubling to 12%^(11)^. Other studies have demonstrated the progression of other ocular diseases as a result of the less frequent ophthalmic care. For example, decreases in both the frequency and quantity of anti-vascular endothelial growth factor (anti-VEGF) injections during the pandemic have been associated with poorer visual acuity^(22^-^24)^.

**Figure 3:**
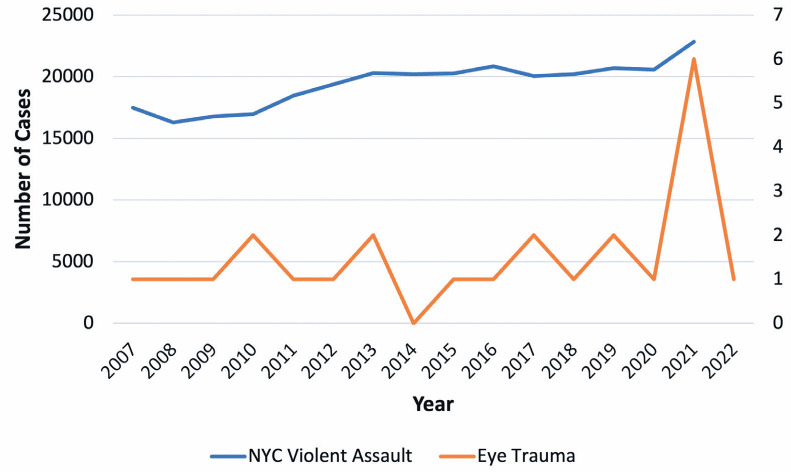
Comparison of the rate of trauma-related eye removal with that of assault/trauma in New York City (2007-2022).

In our study, there was an increase in the rate of procedures performed due to trauma in postpandemic period. The annual rate of eye removals due to trauma was 1.23 before the pandemic and 1.4 in the 5-year period immediately before the pandemic. Following the pandemic, the annual rate increased to 3.0, which is a 243% increase from the rate during the entire prepandemic period and 214% increase from the rate during the 5-year period immediately before the pandemic. To better understand the driving forces behind this in-crease, we analyzed the trends of violent assaults in the city of New York. In total, 22,835 violent assaults took place in 2021, which is a 12% increase from the average of 20,475 assaults per year in the 5 years immediately before the pandemic. Furthermore, in the Bronx, the incidence of felony assaults increased by 23.3% from 2021 to April 2022. This increase coincides with the 428% increase in the rates of trauma-related eye removal procedures in our study^(25)^. The increase in trauma-related eye removals during the pandemic was likely driven by the higher rates of violent assault, which may have escalated due to social stressors such as unemployment, financial hardship, and disruptions to community support systems. Furthermore, the increased trauma--related eye removals may be attributed to the fact that our Level 1 trauma center is located in an area with a high frequency of assault (Figure 3).

A study conducted in Washington, DC that evaluated trauma registry data reported that the overall rates of trauma had decreased by the end of May 2020^(26)^. We noted a similar initial decline in the number of eye removal procedures during this time. In the following year, we noted a sharp increase in the number of eye removal procedures. During the late COVID period in the Bronx, there was an increase in trauma admissions due to more violent mechanisms of injury (e.g., firearm-related injury). This increase coincides with the increased number of eye removal procedures in our study^(27)^. The reasons for these increased trauma rates are outside the scope of this study. Nonetheless, they require further investigation to prevent a similar scenario in the future.

In our study, the increase in rates of eye removal procedures was the highest among Black patients. Before the pandemic, 43.8% of the eye removal procedures in Black patients were due to trauma, 25% were due to a painful blind eye, and another 25% were due to infections. Following the pandemic, 75% of all eye removal procedures in Black patients were due to a painful blind eye, 16.7% were due to trauma, and 16.7% were due to infections. Minority populations are at a greater risk for chronic health conditions than the general population^(28^-^30)^. Given the increase in the incidence of painful blind eyes, preexisting chronic ocular conditions within the Black population may have intensified during the pandemic due to delayed care. Further studies are required to identify the sources of racial disparities and address them. Devising and implementing population--specific measures will help prevent deleterious outcomes that require eye removal.

### Limitations

The main limitations of this study are its retrospective nature and reliance on medical record data. The collected data relies on the completeness and accuracy of the codes recorded by the healthcare practitioners. Another limitation of this study, is the limited sample size and data between the years 2007 and 2022. The first case of COVID-19 infection was reported in Wuhan, China in December 2019. The WHO declared COVID-19 as a pandemic on March 11, 2020, and it formally ended the Public Health Emergency (PHE) state in May 2023. A short time later, the United States ended its PHE state. Thus, our study does not cover the complete pandemic period. Nonetheless, during 2022-2023, 6.6 eye removal procedures were performed, representing a 128% increase in the annual rate from that in the prepandemic period.

The prepandemic years were chosen because they had similarly documented electronic medical records that were available for extraction. Although we noted an increase in the rate of progressive chronic diseases that caused painful blind eyes at other centers, the rates of trauma-related surgeries was higher at our institution. This may be attributed to the fact that our institution is a Level 1 trauma center. Despite these limitations, our study contributes to the existing knowledge regarding the effects of the recent pandemic on eye removal procedures. In the study’s population, the increased rates of trauma-related eye removal correlated with the increa-sed rates of violent assault in the Bronx. Additionally, our data revealed well-studied racial disparities in the presentation of chronic disease, as evidenced by the greatest increase in eye removal procedures among Black patients.

The rates of eye removal procedures at our institution increased during the pandemic. The most common indications for the procedures were painful blind eyes and trauma-related ocular damage. Furthermore, the increased rate of eye removal procedures during the pandemic may be attributed to exacerbations of chronic eye disease due to a delay in ophthalmic care and increased rates of violent assault in New York city (including the Bronx). Our data also demonstrated the greatest increase in the rates of eye removal procedures among the Black population, suggesting racial disparities in access to ophthalmic care during the pandemic. The varied consequences of the recent COVID-19 pandemic on ocular conditions and their management are still being revealed and warrant further studies.
